# Association of pre-clinical depressive symptoms and its plasma metabolomic signature with Parkinson’s disease: a community-based longitudinal study

**DOI:** 10.1038/s41398-026-04364-0

**Published:** 2026-08-17

**Authors:** Xinjie Zhang, Jiao Wang, Sakura Sakakibara, Yang Li, Michelle M. Dunk, Jing Gao, Weili Xu

**Affiliations:** 1 Department of Pediatric Neurosurgery, West China Second University Hospital, Sichuan University, Chengdu, 610041 China; 2 Key Laboratory of Birth Defects and Related Diseases of Women and Children (Sichuan University), Ministry of Education, Chengdu, Sichuan 610041 China; 3 National Clinical Research Center for Geriatrics, West China Hospital, Sichuan University, Chengdu, Sichuan 610041 China; 4 Aging Research Center, Department of Neurobiology, Care Sciences and Society, Karolinska Institutet, Stockholm, 17165 Sweden; 5 School of Public Health, Tianjin Medical University, Tianjin, 300037 China; 6 Thoracic Clinical College, Tianjin Medical University, Tianjin, 300222 China

**Keywords:** Depression, Predictive markers

## Abstract

Accumulating evidence has suggested that depression is associated with an increased risk of Parkinson’s disease (PD), yet whether pre-clinical depressive symptoms in PD development and the potential mediating effects of metabolomic profiles remain unclear. We aimed to examine the association between depressive symptoms and PD risk, evaluate effect modification by genetic susceptibility, and assess metabolomic mediation. This prospective study included 451,922 PD-free participants from the UK Biobank. Depressive symptoms were collected by a 2-item Patient Health Questionnaire (range, 0–6; ≥3 indicates possible depressive disorder) at baseline. Identification of PD was based on medical records. PD-related polygenic risk score (PRS_PD_), which incorporates established genes for PD based on external GWAS meta-analyses, was tertiled as low, moderate, and high. Baseline plasma metabolites were quantified via NMR spectroscopy from blood samples. Data were analyzed using Cox regression. Over a median follow-up of 14.6 years, 3108 (0.7%) participants developed PD. Higher depressive symptom severity showed linear association with PD risk (hazard ratio [HR]: 1.11, 95% confidence interval: 1.07–1.15). Participants with possible depressive disorder had 36% higher PD risk (HR: 1.36, 1.15–1.61). Those with both possible depressive disorder and high genetic risk had the highest PD risk (HR 2.15, 1.74–2.65; *P* for interaction = 0.028). Metabolomic signature, incorporating fatty acids and lipoproteins, accounted for 15% (model-based proportion) of the depressive symptoms-PD association. Pre-clinical depressive symptoms are associated with a moderately increased risk of PD, particularly among people with a high genetic susceptibility to PD. Metabolomic profiles account for a significant portion of this relationship.

**Figure:**
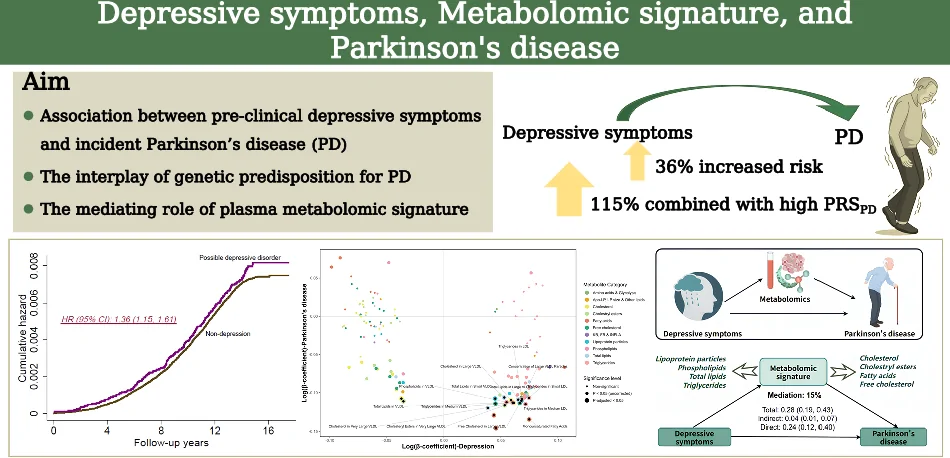

## Introduction

Parkinson’s disease (PD) represents the second most prevalent neurodegenerative disorder worldwide, characterized not only by motor symptoms but also by a range of non-motor manifestations, including depression [1, 2]. With the global burden of PD rising rapidly and no disease-modifying treatments available, identifying modifiable risk factors and early biomarkers has become a public health priority [3, 4]. Indeed, prospective epidemiological studies have consistently reported that individuals with clinically diagnosed depression have an elevated subsequent risk of developing PD [5, 6], though the underlying mechanisms remain incompletely defined.

Most existing evidence derives from studies of clinically diagnosed depression, yet subthreshold depressive symptoms are more common in the general population [7] and may also be relevant to PD risk [8–10]. Clarifying the association between these pre-clinical depressive manifestations and PD could enhance early detection and improve understanding of prodromal disease processes. Genetic factors also play a well-established role in PD susceptibility [11], with recent evidence indicating shared genetic architecture between PD and depression [12]. Yet, whether genetic background modifies the association between depressive symptoms and PD remains largely unexplored [13]. Further elucidation of the potential genetic influence could offer valuable insights into the underlying mechanisms [14].

Metabolomic profiling provides a new perspective for understanding the association between depressive symptoms and PD. Existing research has identified specific metabolic disturbances—such as lipid dysregulation, abnormal fatty acids, and inflammation—that have been independently associated with both depressive symptoms [15] and PD pathogenesis [16]. However, it remains unclear whether these metabolic signatures serve as mediating pathways through which depressive symptoms affect incident PD. Formally validating the mediating role of these metabolites in large-scale population cohort studies is crucial for elucidating the biological link between depressive symptoms and PD.

We aimed to fill these knowledge gaps in the present study using data from a large-scale population-based longitudinal study by: 1) clarifying the extent to which depressive symptoms associate with the risk of developing PD; 2) exploring whether these associations vary according to genetic predisposition for PD (testing for joint-exposure with multiplicative interaction); and 3) evaluating the extent to which plasma metabolomic profiles mediate this association under a model-based causal framework. We hypothesized that higher depressive symptoms would be associated with increased PD risk, that this association would be stronger among those with a high genetic susceptibility, and that part of this association would be statistically mediated by a metabolomic signature involving lipid and inflammation-related pathways.

## Methods

### Study design, setting, and participants

Our study utilized data from the UK Biobank, an ongoing large-scale longitudinal study involving 502,353 UK residents aged 37–73 years [17]. Between 2006 and 2010, participants underwent baseline examinations, including physical and medical assessments and a series of questionnaires covering sociodemographic information and lifestyle behaviors. After excluding 50,431 participants with prevalent PD at study entry (*n* = 901), missing data on depressive symptoms at baseline (*n* = 35,093), or missing PRS_PD_ information (*n* = 16,235), a total of 451,922 participants were included in the current analysis (Supplementary Fig. 1). Among them, 228,585 participants with complete metabolomics data were included in the mediation analysis.

### Assessment of depressive symptoms

The pre-validated Patient Health Questionnaire-2 (PHQ-2) was used to assess depressive symptoms at baseline [18]. The PHQ-2 has been used in observational studies to measure depressive symptoms [18, 19], enabling examination of both symptom severity and the possible presence of depressive disorders. The PHQ-2 has been validated and used in many different populations to screen individuals for depressive tendencies or mild depression rapidly [18, 19]. This 2-item instrument asks about the frequency of depressed mood and anhedonia over the past 2 weeks, with response options being “not at all,” “several days,” “more than half the days,” and “nearly every day,” scored as 0, 1, 2, and 3, respectively. Thus, the PHQ-2 score can range from 0 to 6, and a score ≥ 3 is indicative of possible depressive disorder [19]. The history of clinically diagnosed depression was ascertained by the medical records (ICD-10 code: F32). The PHQ‑2 was used to assess pre‑clinical depressive symptoms. In contrast, clinically diagnosed depression was used only in sensitivity analyses to exclude participants with severe clinical depression at baseline.

### Assessment of Parkinson’s disease

Prevalent and incident PD were ascertained using an algorithm-based method developed by the UK Biobank, which has a positive predictive value of 91% [20]. PD cases were determined based on information from self-reported medical conditions (Field 20002, Code 1262), medical records (ICD-10: G20), and death records from the Hospital Episode Statistics (England), the Scottish Morbidity Record (Scotland), and the Patient Episode Database (Wales). We adopted a conservative approach by using the earliest available date among all sources that indicated a PD diagnosis. This method reduces outcome misclassification due to delayed ascertainment and is consistent with established practice in registry‑based cohort studies. Person-years for each subject were calculated from the recruitment date until the diagnosis of PD, death, loss to follow-up, or end of follow-up (1 October 2023), whichever occurred first.

### Assessment of polygenic risk score

A PD-related polygenic risk score (PRS_PD_) was obtained from the UK Biobank’s Standard PRS Set (October 2022 data release; field ID: 26260) [21]. A total of 26 PD-related single-nucleotide polymorphisms (SNPs) were selected through a meta-analysis of multiple external genome-wide association studies [21]. The PRS_PD_ was calculated by weighted summation according to the strength of each allele’s association with PD, followed by z-transformation [21]. In the current analysis, genetic susceptibility associated with PD was categorized as low, moderate, and high according to the tertiles of PRS_PD_. In the PRS_PD_ analysis, the top 10 genetic ancestry principal components (PCs) were controlled.

### Quantification of plasma metabolites

Detailed information on the UK Biobank procedures for plasma metabolomics data acquisition and processing is available on the UK Biobank website [22]. Briefly, baseline plasma samples were collected between 2006 and 2010 and stored at −80 °C until analysis. Metabolomic profiling was performed in a single wave between June 2019 and April 2020 using Nightingale Health’s high-throughput NMR platform (https://biobank.ndph.ox.ac.uk/showcase/label.cgi?id=220). The biomarker measurements show high repeatability over time, absence of batch effects, and coefficients of variation (CV) below 5% for the majority of biomarkers across spectrometers. A panel of 249 metabolites was quantified from plasma samples collected during the baseline examination, as summarized in Supplementary Table 1. The biomarker data provided by UK Biobank are the values quantified by Nightingale Health’s proprietary software. Biomarker values substantially affected by interfering substances have been removed during the quality control procedures. To account for any residual technical variation (e.g., minor inter-spectrometer differences), all metabolite values were inverse normal transformed for analysis.

### Covariates

Age, sex, race (white vs. non-white), and education (college vs. non-college) were self-reported at baseline. Socioeconomic status was assessed using the Townsend deprivation index (TDI), a measure of socioeconomic deprivation based on neighborhood levels of unemployment, household overcrowding, car non-ownership, and home non-ownership [23]. Higher TDI scores indicate greater socioeconomic deprivation.

Regular physical activity was defined as at least 150 min of moderate activity per week, 75 min of vigorous activity per week, or an equivalent combination [24]. Social contact was assessed based on responses to the question: “How often do you visit friends or family or have them visit you?” Response options included “Almost daily”, “2–4 times a week”, “about once a week”, “about once a month”, “once every few months”, “never or almost never”, “no friends/family outside household”, and “do not know/prefer not to answer” (treated as missing). These responses were further dichotomized as high or low according to the median. Sleep duration was collected via a standardized touch-screen questionnaire at baseline: “About how many hours of sleep do you get in every 24 h? (Please include naps)” and was categorized as < 6 h, 6–8 h, and > 8 h [25].

Hypertension was defined as systolic blood pressure ≥ 140 mm Hg, diastolic blood pressure ≥ 90 mm Hg, use of antihypertensive drugs, or a history of hypertension. Type 2 diabetes was defined as hemoglobin A1c ≥ 6.5%, fasting plasma glucose ≥ 126 mg/dl, having a history of diabetes, or the use of glucose-lowering medications [26]. Cardiovascular disease (CVD), including myocardial infarction, angina, coronary artery disease, congestive heart failure, and atrial fibrillation, was obtained by self-reported medical history and medical records [27].

### Statistical analysis

Baseline characteristics of the study population by depressive symptom status were analyzed using chi-square tests for categorical variables and Student’s t-test for continuous variables.

#### Time-to-event analysis

Cox proportional hazard regression was used to estimate the hazard ratios (HRs) and 95% confidence intervals (CIs) for the incidence of PD associated with depression symptoms, with follow-up time as the time scale. The proportional hazards assumption was verified using Schoenfeld residuals, with no violations detected. Potential nonlinear relationships were examined using 4-knot restricted cubic splines (RCS). Depression symptom score was operationalized as both a continuous and a categorical (no depression [PHQ-2 < 3] vs possible depressive disorder [PHQ-2 ≥ 3]) variable. The continuous PHQ‑2 score was used to test the linear hypothesis—that is, whether PD risk increases incrementally with each unit increase in depressive symptoms. The binary categorization facilitates the interpretation of risk in a clinically meaningful context and allows for more direct comparison with previous studies that focused on clinically significant depression. Models were first basic-adjusted for age, sex, and education level, followed by further adjustment for race, socioeconomic status, BMI, smoking status, alcohol drinking, physical activity, social activity, sleep duration, hypertension, type 2 diabetes, cardiovascular disease, and PRS_PD_. Covariate selection was guided by a directed acyclic graph (DAG; Supplementary Fig. 2), adjusting for confounders (e.g., age, sex, lifestyle, PRS_PD_) in main models while sparing potential mediators (metabolic factors) for mediation analyses; cardiovascular disease and type 2 diabetes were included as confounders based on evidence of shared common causes with both depression and PD. Participants with complete covariate data were included in the Cox regression.

We evaluated the joint effect of depressive symptoms and PRS_PD_ by creating a joint exposure variable and testing for multiplicative interaction via likelihood ratio tests. We also evaluated additive interaction by calculating the relative excess risk due to interaction (RERI) and attributable proportion (AP) using the joint‑effect estimates. Stratified analyses were conducted by sex, age (<65 vs. 65 + years), and other potential effect modifiers (physical activity, sleep duration, cardiovascular disease, type 2 diabetes), with interaction terms included in adjusted models.

#### Mediation analysis

Mediation analysis was performed to assess whether metabolomic profiles explain part of the depression-PD association. Metabolites significantly associated with both depressive symptoms and PD (Benjamini-Hochberg false discovery rate [FDR] *P* < 0.05) were selected. A metabolomic signature score was constructed as a weighted sum of normalized metabolite levels, with weights derived from Cox regression coefficients for PD. We also performed a Principal Component Analysis (PCA) and used PC1 as the metabolomic signature score. We applied a generalized estimation equation framework with nonparametric bootstrapping (500 replicates) to estimate direct and indirect effects. Path a (depressive symptoms → metabolomic signature) was estimated using multinomial logistic regression, and path b (metabolomic signature → PD) using Weibull survival models. Indirect effects were calculated as the product of paths a and b, with the mediation proportion derived as indirect/total effect. This analysis provides a statistical decomposition of the observed association under the model’s assumptions, including the critical assumption of correct temporal ordering. All sub-models included the same full set of covariates listed in the main analyses. No exposure-mediator interaction was modeled in the analysis. The proportional hazards assumption was assessed and met for the mediator in the path b model, with no violations detected (*P* = 0.549).

Sensitivity analyses included: (1) excluding participants aged < 50 years; (2) excluding those with baseline clinical depression; (3) using Poisson regression due to low outcome incidence; (4) excluding PD cases within the first 3 years and 5 years of follow-up to address potential reverse causality; (5) imputing missing values for covariates using multiple imputation by chained equations; (6) excluding participants with prevalent or incident other Parkinsonian syndromes (ICD code: G21, G22, and G23); and using age as time-scale in the Cox regression models.

Alpha was set at 0.05 for all analyses. All statistical analyses were performed using Stata SE 16.0 (Stata Corp, College Station, Texas).

## Results

### Baseline characteristics of the study population

The mean age at baseline was 56.6 ± 8.1 years, and 54.0% of participants were female. Among all participants, 26,743 (5.9%) individuals had a possible depressive disorder. Compared to participants without depression, those with possible depressive disorder were more likely to be younger, female, and of a non-white race; to have lower TDI and BMI, a lower education level, irregular physical activity, a higher level of social contact, and less sleep duration; and to be non-drinkers and current smokers. Individuals with depressive symptoms reported a higher frequency of social contact, which may reflect differences in reporting (i.e., individuals with depressive symptoms may perceive or report social interactions differently than the general population) or may indicate that the PHQ-2 scale captures a subgroup whose social functioning remains normal despite the presence of mood symptoms. In addition, those with possible depressive disorder tended to have a history of type 2 diabetes, cardiovascular disease, and clinically diagnosed depression (Table 1). There was no significant difference between participants with complete covariates and those missing any information (Supplementary Table 2).

**Table 1:** Baseline characteristics of the study populations by depressive symptoms (*N* = 451,922).

Characteristics	Possible depressive disorder (*n* = 26,743)	No depression (*n* = 425,179)	*P*-value
Follow-up time (year)	14.55 (13.67, 15.32)	14.61 (13.84, 15.33)	-
Age (year)	54.25 ± 7.94	56.71 ± 8.07	<0.001
Female	15,319 (57.28)	228,808 (53.81)	<0.001
Race-White	22,575 (84.82)	389,205 (91.81)	<0.001
Townsend deprivation index	−2.27 (−3.70, 0.25)	−0.55 (−2.92, 2.74)	<0.001
Education (college)	6602 (25.12)	143,567 (34.08)	<0.001
BMI (kg/m^2^)	27.31 ± 4.68	28.58 ± 5.80	<0.001
Alcohol drinking			<0.001
Never	2231 (8.37)	16,383 (3.86)	
Former drinker	1937 (7.27)	13,937 (3.28)	
Current drinker	22,493 (84.37)	394,594 (92.86)	
Smoking status			<0.001
Never	13,307 (49.99)	233,271 (55.03)	
Former smoker	8018 (30.12)	149,276 (35.22)	
Current smoker	5295 (19.89)	41,350 (9.75)	
Regular physical activity	14,678 (63.26)	287,694 (72.59)	<0.001
High level of social contact	16,451 (62.16)	241,745 (57.15)	<0.001
Sleep duration			<0.001
<6 h	13,190 (50.13)	293,327 (69.25)	
6–8 h	9991 (37.97)	99,148 (23.41)	
>8 h	3129 (11.89)	31,098 (7.34)	
Hypertension	14,580 (54.52)	230,423 (54.19)	0.301
Type 2 Diabetes	2436 (9.11)	20,984 (4.94)	<0.001
Cardiovascular disease	2612 (9.77)	27,472 (6.46)	<0.001
History of depression	7648 (28.65)	28,345 (6.67)	<0.001
PRS_PD_	−0.14 (−0.83, 0.55)	−0.15 (−0.83, 0.54)	0.545

Data were presented as mean ± standard deviation for continuous variables and number (percentage) for categorical variables.

*BMI* body mass index, *PRS*_*PD*_ polygenic risk score for Parkinson’s disease.

Missing data: Education = 4390; Race = 1384; Townsend deprivation index = 555; Smoking = 1405; Alcohol consumption = 341; BMI = 1640; Physical activity = 32,394; Social contact = 2455; Sleep duration = 2039.

### Association between depressive symptoms and incident Parkinson’s disease

During a median follow-up of 14.6 years (interquartile range: 13.8 to 15.3 years; totaling 3,240,608 person-years), 3108 (0.7%) incident cases of PD were identified, including 2914 among participants free of possible depressive disorder and 194 among those with possible depressive disorder.

**Fig. 1:**
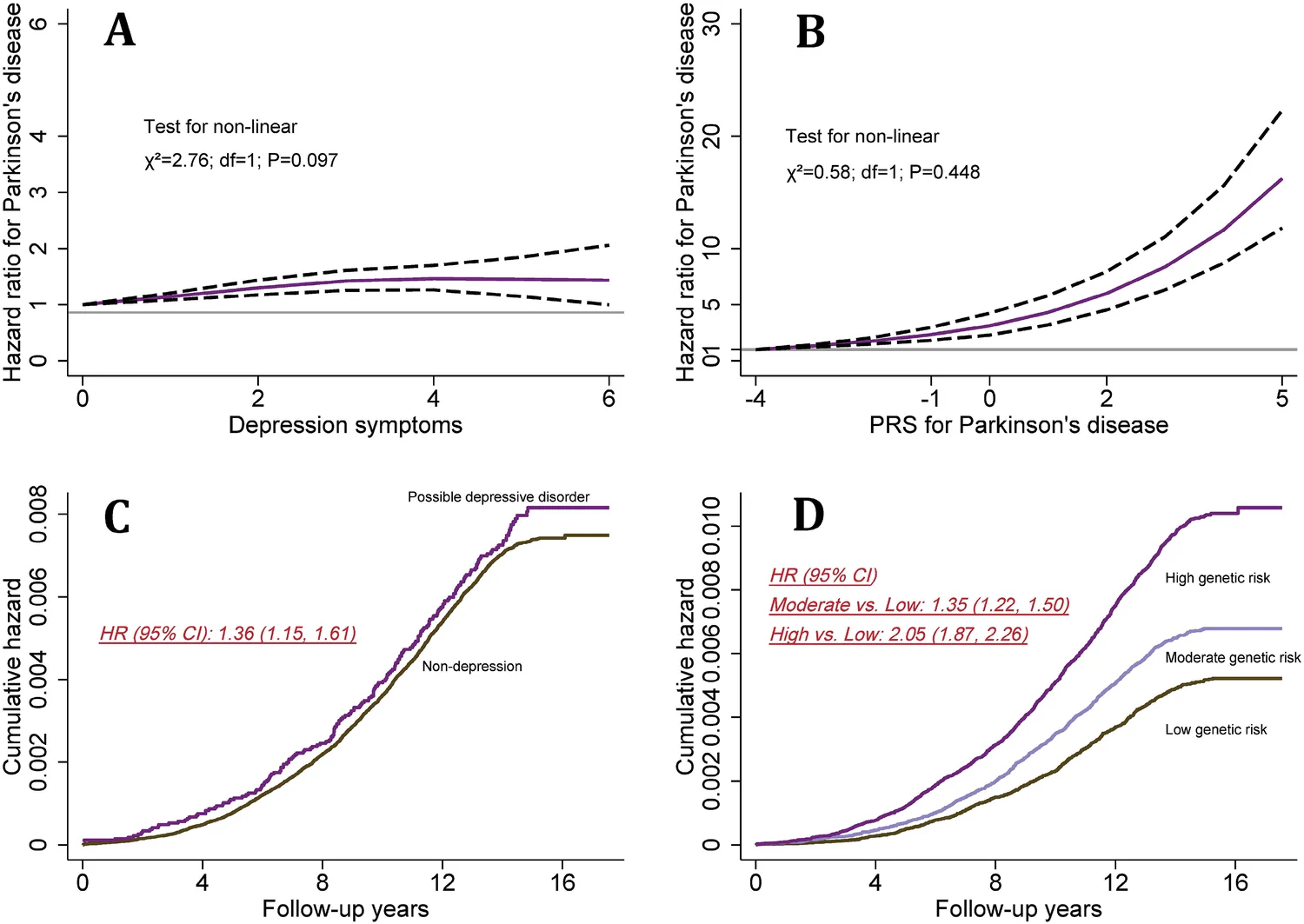
Association between depressive symptoms and risk of incident Parkinson’s disease. **A** and **B** Associations between continuous depressive symptoms and PRS_PD_ with Parkinson's disease; Panel **C** and **D** Associations between categorical depressive symptoms and PRS_PD_ with Parkinson's disease. Models were adjusted for age, sex, education, race, socioeconomic status, body mass index, smoking status, alcohol drinking, physical activity, social activity, sleep duration, hypertension, diabetes, and cardiovascular disease. Models for depression symptoms were additionally adjusted for PRS_PD_. Models for PRS_PD_ were additionally adjusted for depression symptoms.

Higher depressive symptoms score was related to a higher risk of PD (HR [95% CI]: 1.13 [1.10, 1.17] in the basic-adjusted model and 1.11 [1.07, 1.15] in the multi-adjusted model; Table 2). Participants with possible depressive disorder had a higher risk of PD compared to those without (HR [95% CI]: 1.53 [1.32, 1.78] in the basic-adjusted model; 1.36 [1.15, 1.61] in the multi-adjusted model) (Table 2 and Fig. 1C). No significant nonlinear association was observed (*P*_*non-linear*_ > 0.05, Fig. 1A).

**Table 2:** Incident Parkinson’s disease in relation to depressive symptoms and genetic risk factors: results from Cox regressions.

	No. of participants	Parkinson’s disease		
		No. of cases	HR (95% CI)^a^	HR (95% CI)^b^
**Depressive symptoms**				
Continuous	451,922	3108	1.13 (1.10, 1.17)	1.11 (1.07, 1.15)
Categorical				
No depression	425,179	2914	Reference	Reference
Possible depressive disorder	26,743	194	1.53 (1.32, 1.78)	1.36 (1.15, 1.61)
**PRS**_**PD**_				
Continuous (per 1-SD increase)	451,922	3108	1.40 (1.35, 1.45)	1.41 (1.36, 1.47)
Categorical				
Low	150,641	720	Reference	Reference
Moderate	150,642	950	1.34 (1.22, 1.48)	1.35 (1.22, 1.50)
High	150,639	1438	2.03 (1.86, 2.22)	2.05 (1.87, 2.26)

*PRS*_*PD*_ polygenic risk score for Parkinson’s disease.

^a^Models were adjusted for age, sex, and education.

^b^Models were adjusted for age, sex, education, race, socioeconomic status, body mass index, smoking status, alcohol drinking, physical activity, social activity, sleep duration, hypertension, diabetes, and cardiovascular disease. Models for depression symptoms were additionally adjusted for PRS_PD_. Models for PRS_PD_ were additionally adjusted for depression symptoms.

### Role of genetic risk factors

A linear relationship was observed between genetic susceptibility and PD risk (Table 2 and Fig. 1B and D), with high PRS_PD_ associated with more than doubled risk (HR 2.05, 95% CI 1.87–2.26). In joint-effect analysis (Supplementary Table 3), participants with both depressive disorder and high PRS_PD_ exhibited the highest PD risk (HR 2.15, 95% CI 1.74–2.65), with significant multiplicative interaction (*P for interaction* = 0.028). However, we failed to find a significant additive interaction (RERI [95% CI]: −0.049 [−0.737 to 0.638]; AP [95% CI]: −0.021 [−0.316 to 0.274]). In stratified analyses (Supplementary Table 3), possible depressive disorder was associated with a higher PD risk in both low PRS (HR 1.72, 95% CI 1.25–2.38) and high PRS (HR 1.31, 95% CI 1.02–1.69) groups.

### Metabolomic mediation effect

**Fig. 2:**
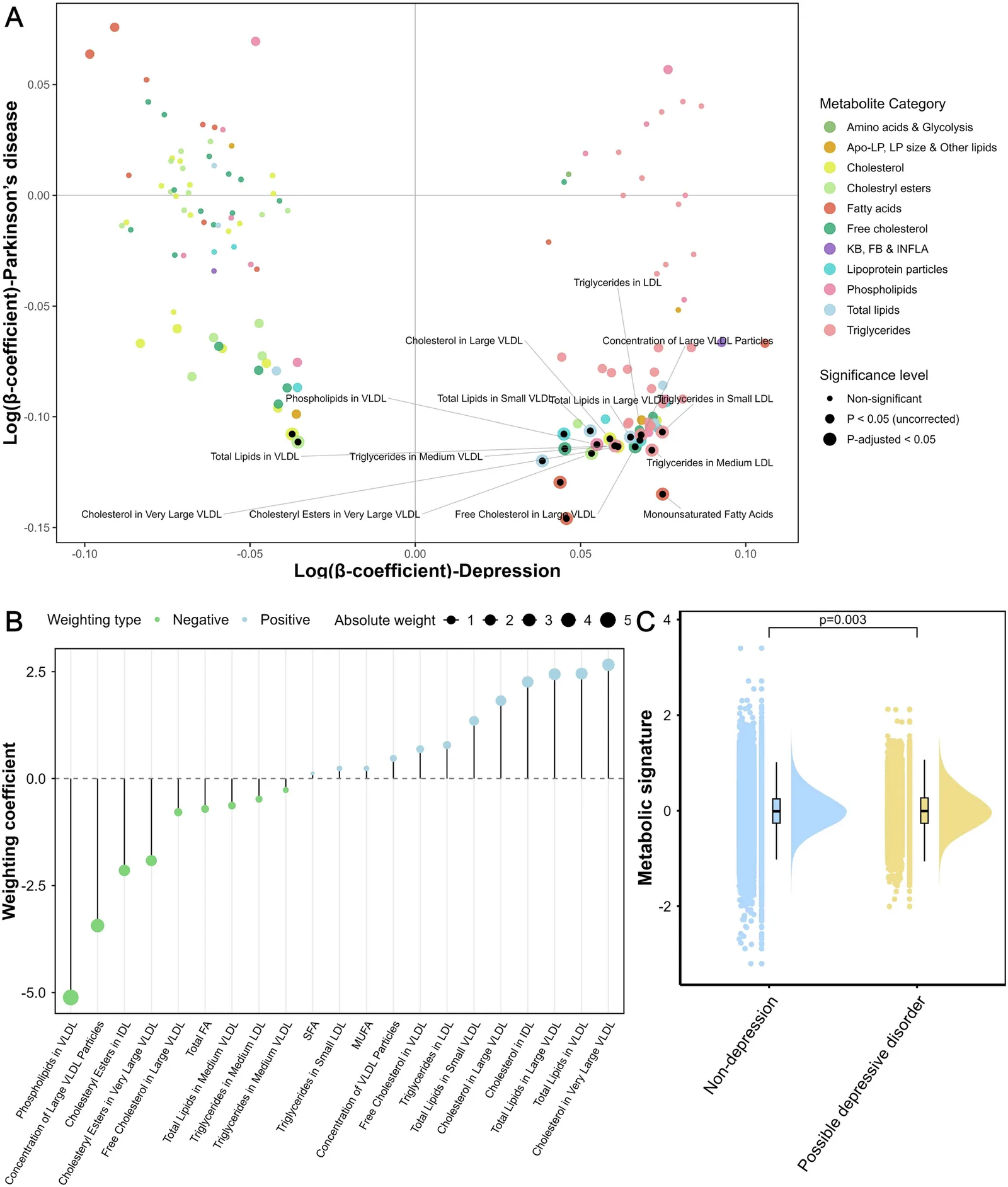
Association between plasma metabolomic signature, pre-clinical depressive symptoms, and Parkinson’s disease. **A** showing associations of metabolites with depressive symptoms (x-axis) and Parkinson’s disease (y-axis). Each bubble represents a metabolite, colored by biochemical category and formatted by significance: solid (FDR < 0.05), patterned (nominal *P* < 0.05), open (non-significant). **B** displays the Cox regression-derived coefficients (β) for the association between each of the 21 metabolites and incident Parkinson’s disease. These coefficients were used to weight the respective metabolite levels in calculating the individual Metabolomic Signature Score as a weighted sum. **C** shows the distribution of the Metabolomic Signature Score by depressive disorder status. VLDL very low-density lipoprotein, LDL low-density lipoprotein, Apo-LP apolipoproteins, LP lipoprotein, KB ketone bodies, FB fluid balance, INFLA inflammatory markers.

**Fig. 3:**
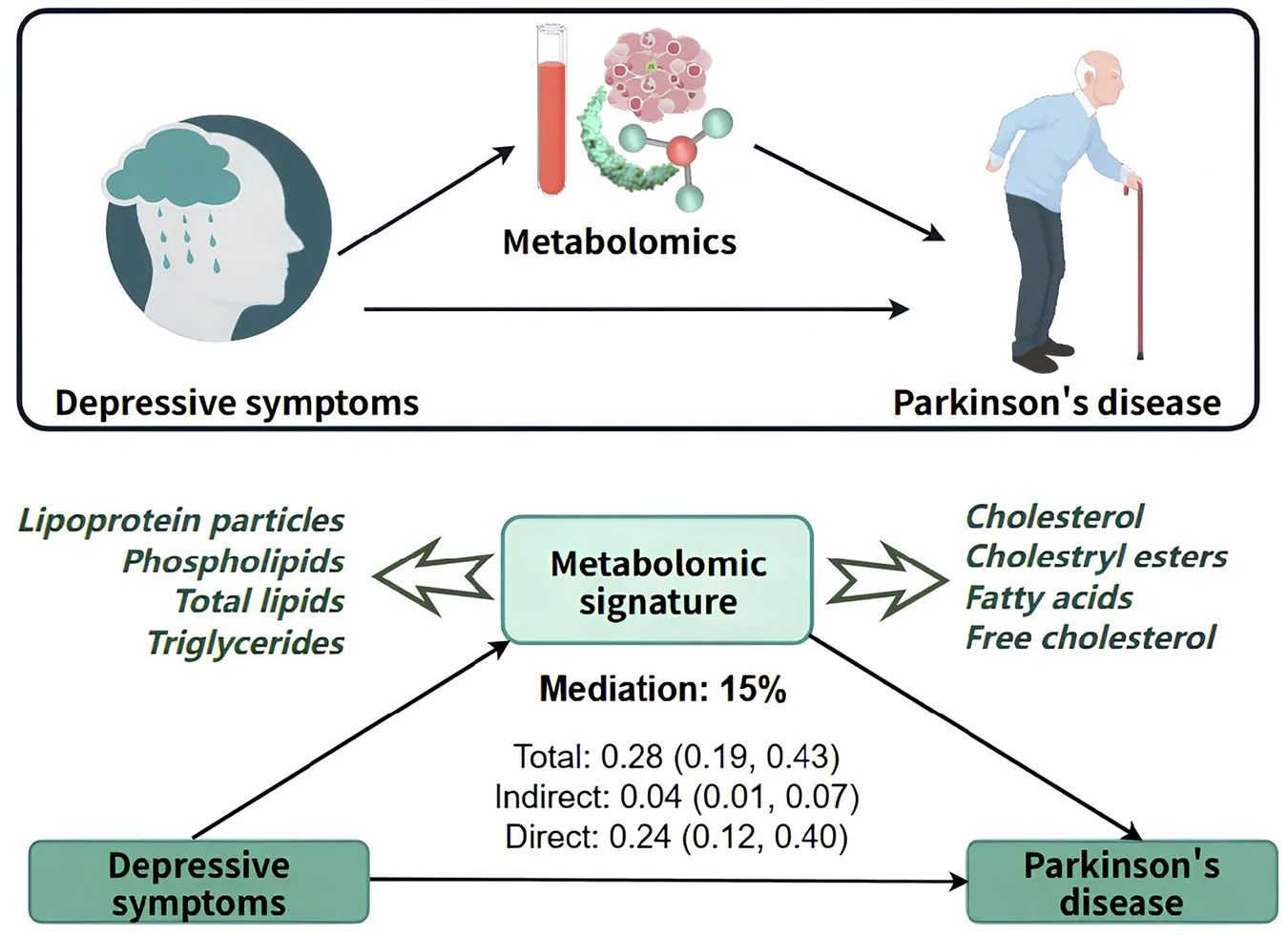
Mediation role of plasma metabolomic signature in the association between pre-clinical depressive symptoms and incident Parkinson’s disease. The boxed diagram illustrates the hypothesized mediation model for the relationship between depressive symptoms, metabolites, and Parkinson’s disease. Plasma metabolomic signatures were operationalized as 3-level categorical variables (low, medium, high) by merging the middle three quintiles (Q2-Q4). The paths in the model were estimated as follows: the a-path (depressive symptoms→metabolites) was modeled using multinomial logistic regression; the b-path (metabolites→Parkinson’s disease) and the c’-path (direct effect of depressive symptoms→Parkinson’s disease) were jointly estimated using a survival model assuming a Weibull distribution. Models were all adjusted for age, sex, education, race, socioeconomic status, body mass index, smoking status, alcohol drinking, physical activity, social activity, sleep duration, hypertension, diabetes, and cardiovascular disease.

Of the 249 plasma metabolites, 21 demonstrated significant associations with both depressive symptoms and incident PD (Fig. 2). These substances primarily comprise cholesterol, cholesterol esters, fatty acids, free cholesterol, lipoprotein particles, phospholipids, total lipids, and triglycerides. The metabolomic signature score constructed from these metabolites significantly mediated the depression-PD association (Fig. 3 and Supplementary Table 4), explaining 15% of the total effect (indirect effect: 0.04, 95% CI 0.01–0.07; direct effect: 0.24, 95% CI 0.12–0.40). To examine the robustness, we performed a sensitivity mediation analysis using the first principal component (explaining 82.47% of the variance) derived from the 21 identified metabolites as an alternative, unsupervised metabolic score. The mediation proportion obtained with this PC-based score was 22%, which aligns with our primary result (Supplementary Table 5).

### Supplementary analysis

There were no significant interactions (all *P for interaction* > 0.05) between depressive symptoms and sex, age, physical activity, sleep duration, cardiovascular disease, or type 2 diabetes (Supplementary Table 6). The associations remained robust in sensitivity analyses excluding 52,480 participants aged < 50 years, those with baseline depression (*n* = 18,921), 68 incident PD cases that occurred during the first 3 years and 5 years of the follow-up, 396 participants with prevalent or incident other Parkinsonian syndromes, after imputing missing values for covariates, and when using alternative statistical models or treating age as a time scale in Cox regression models (Supplementary Tables 7–14).

## Discussion

In this large-scale community-based cohort study, we found that pre-clinical depressive symptoms were associated with a higher risk of PD during the 17-year follow-up. We also found that the higher risk of PD in relation to possible depressive disorder was more pronounced among participants with a high genetic susceptibility to PD. Notably, the relationship was partially explained by plasma metabolomic signatures.

Our results extend previous evidence linking clinical depression with PD risk by focusing on pre-clinical depressive symptoms, which are more prevalent in the general population yet have been less studied in relation to PD. While previous investigations have primarily examined clinically diagnosed depression [5, 6], our study demonstrates that even subthreshold depressive symptoms, as measured by the standardized PHQ-2, are associated with a higher risk of PD. This observation aligns with emerging evidence suggesting that depressive manifestations may appear years before motor symptom onset in PD [8–10]. However, unlike previous studies limited by small sample sizes [9] or non-validated instruments [28], our findings from a large population-based cohort provide robust evidence for the temporal relationship between pre-clinical depressive symptoms and subsequent PD development.

The significant multiplicative interaction between depressive symptoms and genetic susceptibility represents another key finding. The latest genome-wide association studies have revealed that genetic variants explain only 16% of PD risk [11], and modifiable environmental risk factors account for a relatively small proportion of PD risk [29]. These modest effect sizes may indicate that the gene-environment interactions play a considerable role in the remaining risk and could provide insight into PD pathobiology [13]. We found that the risk of developing PD was significantly higher among those with both possible depressive disorder and high genetic risk. This statistical association lacks a clear biological mechanism, but several explanatory mechanisms may exist. First, individuals with high genetic risk may exhibit underlying neuroinflammation, and depressive symptoms could exacerbate inflammation, thereby accelerating PD progression [30]. Second, genetic risk may reduce neuroplasticity in PD-related brain regions, while depression further suppresses neuroprotection (e.g., BDNF) [31], limiting the brain’s capacity to compensate for early dopaminergic neuronal damage. Although a significant multiplicative interaction was observed, an analysis on the additive scale did not reveal a significant additive effect. This distinction suggests that while individuals with both risk factors have substantially elevated relative risk (HR 2.15), the absolute excess risk attributable to their co-occurrence may not exceed the sum of their independent effects. This pattern indicates that, on the relative scale, depressive symptoms and genetic susceptibility modify each other’s effects, which is relevant for etiologic understanding and risk stratification. From a public health perspective, even in the absence of a significant additive interaction, this subgroup remains a high-priority population for potential monitoring or intervention, as their absolute risk is substantially elevated. Whether prevention or mitigation of depressive symptoms could modify genetic risk for PD needs to be addressed in more depth in future studies.

Notably, our mediation analysis revealed that plasma metabolomic profiles may partially account for 15% of the association between depressive symptoms and PD, under the assumptions of the mediation model and given the cross‑sectional measurement of the mediator. The metabolites contributing to this signature were predominantly related to lipoprotein composition and cholesterol metabolism. These metabolic features have been previously implicated in both depression-related pathophysiology and PD [15, 16], supporting their role as plausible biological intermediaries. The observed mediation effect provides empirical evidence that systemic metabolic disturbances—particularly in lipid-related pathways—may partly underlie the established epidemiological link between depressive symptoms and PD. However, it is important to note that the result should be interpreted as a statistical illustration of a plausible biological pathway, rather than definitive causal mediation.

Several biological mechanisms may explain the association between depressive symptoms and PD. First, PD-associated depletion of dopaminergic neurons in the substantia nigra reveals that depressive symptoms may be part of the pathophysiologic process that leads to PD, rather than just a simple mood disorder [32]. Second, neuroinflammation may be a key nexus. Chronic low-grade inflammation induced by depressive symptoms [33] may accelerate the accumulation of pathologies associated with the onset of PD, such as elevated proinflammatory cytokines (e.g., IL-6, TNF-α) promoting microglia activation, oxidative stress in dopaminergic neurons, and α-synuclein aggregation [34]. In addition, genome-wide association studies have identified shared genetic risk loci for PD and depression [12]. This evidence also supports our finding of the significant interaction between depressive symptoms and PRS_PD_ in the development of PD. It is notable that the long prodromal phase of PD often delays formal diagnosis. Therefore, screening for depressive symptoms in the clinical setting could help in the early detection and treatment of PD.

Strengths of this study include the community-based design with a large sample size and a rigorous data collection procedure. In addition, we were able to consider a wide range of confounders by leveraging the extensive information available in the UK Biobank, such as genetic susceptibility to PD according to a comprehensive PRS not readily available in other studies. Nonetheless, some limitations should be pointed out. First, UK Biobank participants were volunteers and predominantly of white ethnicity, likely representing a healthier subset of the general population [35, 36]. Consequently, this may have led to an underestimation of the association between depressive symptoms and PD incidence. Furthermore, caution is needed when extrapolating our results to populations of non-white European ancestry. Second, the use of links to electronic health records for diagnosis may have high specificity but low sensitivity [37], and early-stage cases of PD may not have been captured, potentially attenuating the observed relationship between depressive symptoms and PD risk. Although we cannot rule out the possibility of differential detection between participants with and without depressive symptoms, any resulting bias would, if present, complicate the interpretation of the observed hazards. Third, considering that PD has a prolonged preclinical phase, the presence and severity of depressive symptoms may be biased among participants with preclinical or prodromal PD [38]. Although we performed progressively stringent sensitivity analyses excluding participants with likely preclinical/prodromal PD (i.e., incident PD cases occurring during the first three and then five years of follow-up)—with the association remaining significant—the prodromal phase of PD may extend for a decade or more. Thus, while our analyses strengthen the temporal evidence for an association, we cannot definitively determine whether pre-clinical depressive symptoms represent an independent risk factor for PD or an early non-motor manifestation of underlying disease processes. The observed association should therefore be interpreted as reflecting an early clinical signal of impending PD rather than conclusive evidence for a causal risk factor. Fourth, depressive symptoms were assessed using the brief PHQ-2 questionnaire, which captures core symptoms (depressed mood and anhedonia) but fails to encompass the full spectrum, severity, and chronic nature of depression. This brevity likely introduces non-differential misclassification, meaning our current hazard estimates may be conservatively biased toward the null, potentially underestimating the true magnitude of the association. Furthermore, symptom reporting may vary across age groups and cultural backgrounds. In volunteer-based cohorts, potential confounding biases associated with help-seeking behavior cannot be entirely ruled out. Finally, our mediation analysis is hypothesis-generating and carries important limitations. The estimated proportion mediated is model‑based and relies on strong assumptions, especially the temporal ordering from depressive symptoms to metabolites—a sequence we cannot verify with single‑time‑point data. Our mediation analysis relies on single-time-point metabolomic data. Because we cannot track longitudinal metabolic trajectories, our results reflect a snapshot of baseline metabolic vulnerability rather than dynamic changes over the prolonged follow-up. External validation with repeated measures is needed. Furthermore, selecting metabolites associated with both exposure and outcome in the same sample may overfit the mediation estimate. Therefore, the result should be interpreted as a statistical illustration of a plausible pathway, not as causal evidence. External validation with repeated measures is needed. In addition, the observed metabolomic signature is likely influenced by factors not fully captured in our models, such as detailed diet and medication use (e.g., statins). These could act as unmeasured confounders of the mediator-outcome relationship and partially bias the estimated mediation effect.

## Conclusion

Our findings indicate that pre-clinical depressive symptoms are associated with a higher risk of Parkinson’s disease, with this relationship being partially explained by metabolomic alterations and modified by genetic susceptibility. These results highlight the potential value of integrated assessment of depressive symptoms, metabolic profiles, and genetic risk, which may help identify individuals at elevated PD risk for closer monitoring.

## Supplementary information

Supplementary

## Data Availability

The datasets generated and analyzed during the current study are available in the UK Biobank repository, http://www.ukbiobank.ac.uk. The derived variables used in this study (e.g., the polygenic risk score and the metabolomic signature score) were constructed within the UK Biobank framework following the methods described in this paper and are not directly distributable. However, they can be reproduced by approved researchers using the provided algorithms on the underlying UK Biobank data.
