# Supplementary material for: Association of pre-clinical depressive symptoms and its plasma metabolomic signature with Parkinson’s disease: a community-based longitudinal study
Source: Transl Psychiatry. 2026 Aug 17;16:634. doi: 10.1038/s41398-026-04364-0 (PMC13754773; doi:10.1038/s41398-026-04364-0)
Supplement: Supplementary file 1 [file 41398_2026_4364_MOESM1_ESM.docx]

**Baseline included participants, n=502,353**

**Analytical sample, n=451,922**

**Baseline NMR subsample with complete metabolites**

**n = 228,585**

No depression

n = 425,179

PD: 2,914

Possible depressive disorder

n =26,743

PD: 194

We excluded 50,431 persons due to prevalent Parkinson’s disease (n=901), missing depressive symptoms data (n=35,093), and missing PRS_PD_ information (n=16,235).

No depression

n = 215,114

PD: 1,526

Possible depressive disorder

n =13,471

PD: 104

**Supplementary Figure 1.** Flowchart of the study population.

Abbreviations: PRS_PD_ = polygenic risk score for Parkinson’s disease.

**Supplementary Table 1.** NMR metabolites and corresponding field ID in UK Biobank.

| **Field_ID** | **Description** | **Abbreviation** |
| --- | --- | --- |
| 23474 | 3-Hydroxybutyrate | BHBA |
| 23475 | Acetate | Acetate |
| 23476 | Acetoacetate | Acetoacetate |
| 23477 | Acetone | Acetone |
| 23460 | Alanine | Ala |
| 23479 | Albumin | Albumin |
| 23440 | Apolipoprotein A1 | ApoA1 |
| 23439 | Apolipoprotein B | ApoB |
| 23441 | Apolipoprotein B to Apolipoprotein A1 ratio | ApoB/ApoA1 |
| 23433 | Average Diameter for HDL Particles | HDL size |
| 23432 | Average Diameter for LDL Particles | LDL size |
| 23431 | Average Diameter for VLDL Particles | VLDL size |
| 23484 | Cholesterol in Chylomicrons and Extremely Large VLDL | XXL-VLDL-C |
| 23526 | Cholesterol in IDL | IDL-C |
| 23561 | Cholesterol in Large HDL | L-HDL-C |
| 23533 | Cholesterol in Large LDL | L-LDL-C |
| 23498 | Cholesterol in Large VLDL | L-VLDL-C |
| 23568 | Cholesterol in Medium HDL | M-HDL-C |
| 23540 | Cholesterol in Medium LDL | M-LDL-C |
| 23505 | Cholesterol in Medium VLDL | M-VLDL-C |
| 23575 | Cholesterol in Small HDL | S-HDL-C |
| 23547 | Cholesterol in Small LDL | S-LDL-C |
| 23512 | Cholesterol in Small VLDL | S-VLDL-C |
| 23554 | Cholesterol in Very Large HDL | XL-HDL-C |
| 23491 | Cholesterol in Very Large VLDL | XL-VLDL-C |
| 23519 | Cholesterol in Very Small VLDL | XS-VLDL-C |
| 23580 | Cholesterol to Total Lipids in Chylomicrons and Extremely Large VLDL percentage | XXL-VLDL-C/TL (%) |
| 23610 | Cholesterol to Total Lipids in IDL percentage | IDL-C/TL (%) |
| 23635 | Cholesterol to Total Lipids in Large HDL percentage | L-HDL-C/TL (%) |
| 23615 | Cholesterol to Total Lipids in Large LDL percentage | L-LDL-C/TL (%) |
| 23590 | Cholesterol to Total Lipids in Large VLDL percentage | L-VLDL-C/TL (%) |
| 23640 | Cholesterol to Total Lipids in Medium HDL percentage | M-HDL-C/TL (%) |
| 23620 | Cholesterol to Total Lipids in Medium LDL percentage | M-LDL-C/TL (%) |
| 23595 | Cholesterol to Total Lipids in Medium VLDL percentage | M-VLDL-C/TL (%) |
| 23645 | Cholesterol to Total Lipids in Small HDL percentage | S-HDL-C/TL (%) |
| 23625 | Cholesterol to Total Lipids in Small LDL percentage | S-LDL-C/TL (%) |
| 23600 | Cholesterol to Total Lipids in Small VLDL percentage | S-VLDL-C/TL (%) |
| 23630 | Cholesterol to Total Lipids in Very Large HDL percentage | XL-HDL-C/TL (%) |
| 23585 | Cholesterol to Total Lipids in Very Large VLDL percentage | XL-VLDL-C/TL (%) |
| 23605 | Cholesterol to Total Lipids in Very Small VLDL percentage | XS-VLDL-C/TL (%) |
| 23485 | Cholesteryl Esters in Chylomicrons and Extremely Large VLDL | XXL-VLDL-CE |
| 23418 | Cholesteryl Esters in HDL | HDL-CE |
| 23527 | Cholesteryl Esters in IDL | IDL-CE |
| 23562 | Cholesteryl Esters in Large HDL | L-HDL-CE |
| 23534 | Cholesteryl Esters in Large LDL | L-LDL-CE |
| 23499 | Cholesteryl Esters in Large VLDL | L-VLDL-CE |
| 23417 | Cholesteryl Esters in LDL | LDL-CE |
| 23569 | Cholesteryl Esters in Medium HDL | M-HDL-CE |
| 23541 | Cholesteryl Esters in Medium LDL | M-LDL-CE |
| 23506 | Cholesteryl Esters in Medium VLDL | M-VLDL-CE |
| 23576 | Cholesteryl Esters in Small HDL | S-HDL-CE |
| 23548 | Cholesteryl Esters in Small LDL | S-LDL-CE |
| 23513 | Cholesteryl Esters in Small VLDL | S-VLDL-CE |
| 23555 | Cholesteryl Esters in Very Large HDL | XL-HDL-CE |
| 23492 | Cholesteryl Esters in Very Large VLDL | XL-VLDL-CE |
| 23520 | Cholesteryl Esters in Very Small VLDL | XS-VLDL-CE |
| 23416 | Cholesteryl Esters in VLDL | VLDL-CE |
| 23581 | Cholesteryl Esters to Total Lipids in Chylomicrons and Extremely Large VLDL percentage | XXL-VLDL-CE/TL (%) |
| 23611 | Cholesteryl Esters to Total Lipids in IDL percentage | IDL-CE/TL (%) |
| 23636 | Cholesteryl Esters to Total Lipids in Large HDL percentage | L-HDL-CE/TL (%) |
| 23616 | Cholesteryl Esters to Total Lipids in Large LDL percentage | L-LDL-CE/TL (%) |
| 23591 | Cholesteryl Esters to Total Lipids in Large VLDL percentage | L-VLDL-CE/TL (%) |
| 23641 | Cholesteryl Esters to Total Lipids in Medium HDL percentage | M-HDL-CE/TL (%) |
| 23621 | Cholesteryl Esters to Total Lipids in Medium LDL percentage | M-LDL-CE/TL (%) |
| 23596 | Cholesteryl Esters to Total Lipids in Medium VLDL percentage | M-VLDL-CE/TL (%) |
| 23646 | Cholesteryl Esters to Total Lipids in Small HDL percentage | S-HDL-CE/TL (%) |
| 23626 | Cholesteryl Esters to Total Lipids in Small LDL percentage | S-LDL-CE/TL (%) |
| 23601 | Cholesteryl Esters to Total Lipids in Small VLDL percentage | S-VLDL-CE/TL (%) |
| 23631 | Cholesteryl Esters to Total Lipids in Very Large HDL percentage | XL-HDL-CE/TL (%) |
| 23586 | Cholesteryl Esters to Total Lipids in Very Large VLDL percentage | XL-VLDL-CE/TL (%) |
| 23606 | Cholesteryl Esters to Total Lipids in Very Small VLDL percentage | XS-VLDL-CE/TL (%) |
| 23473 | Citrate | Citrate |
| 23404 | Clinical LDL Cholesterol | Clinical LDL-C |
| 23481 | Concentration of Chylomicrons and Extremely Large VLDL Particles | XXL-VLDL-P |
| 23430 | Concentration of HDL Particles | HDL-P |
| 23523 | Concentration of IDL Particles | IDL-P |
| 23558 | Concentration of Large HDL Particles | L-HDL-P |
| 23530 | Concentration of Large LDL Particles | L-LDL-P |
| 23495 | Concentration of Large VLDL Particles | L-VLDL-P |
| 23429 | Concentration of LDL Particles | LDL-P |
| 23565 | Concentration of Medium HDL Particles | M-HDL-P |
| 23537 | Concentration of Medium LDL Particles | M-LDL-P |
| 23502 | Concentration of Medium VLDL Particles | M-VLDL-P |
| 23572 | Concentration of Small HDL Particles | S-HDL-P |
| 23544 | Concentration of Small LDL Particles | S-LDL-P |
| 23509 | Concentration of Small VLDL Particles | S-VLDL-P |
| 23551 | Concentration of Very Large HDL Particles | XL-HDL-P |
| 23488 | Concentration of Very Large VLDL Particles | XL-VLDL-P |
| 23516 | Concentration of Very Small VLDL Particles | XS-VLDL-P |
| 23428 | Concentration of VLDL Particles | VLDL-P |
| 23478 | Creatinine | Creatinine |
| 23443 | Degree of Unsaturation | Unsaturation |
| 23450 | Docosahexaenoic Acid | DHA |
| 23457 | Docosahexaenoic Acid to Total Fatty Acids percentage | DHA/TFA (%) |
| 23486 | Free Cholesterol in Chylomicrons and Extremely Large VLDL | XXL-VLDL-FC |
| 23422 | Free Cholesterol in HDL | HDL-FC |
| 23528 | Free Cholesterol in IDL | IDL-FC |
| 23563 | Free Cholesterol in Large HDL | L-HDL-FC |
| 23535 | Free Cholesterol in Large LDL | L-LDL-FC |
| 23500 | Free Cholesterol in Large VLDL | L-VLDL-FC |
| 23421 | Free Cholesterol in LDL | LDL-FC |
| 23570 | Free Cholesterol in Medium HDL | M-HDL-FC |
| 23542 | Free Cholesterol in Medium LDL | M-LDL-FC |
| 23507 | Free Cholesterol in Medium VLDL | M-VLDL-FC |
| 23577 | Free Cholesterol in Small HDL | S-HDL-FC |
| 23549 | Free Cholesterol in Small LDL | S-LDL-FC |
| 23514 | Free Cholesterol in Small VLDL | S-VLDL-FC |
| 23556 | Free Cholesterol in Very Large HDL | XL-HDL-FC |
| 23493 | Free Cholesterol in Very Large VLDL | XL-VLDL-FC |
| 23521 | Free Cholesterol in Very Small VLDL | XS-VLDL-FC |
| 23420 | Free Cholesterol in VLDL | VLDL-FC |
| 23582 | Free Cholesterol to Total Lipids in Chylomicrons and Extremely Large VLDL percentage | XXL-VLDL-FC/TL (%) |
| 23612 | Free Cholesterol to Total Lipids in IDL percentage | IDL-FC/TL (%) |
| 23637 | Free Cholesterol to Total Lipids in Large HDL percentage | L-HDL-FC/TL (%) |
| 23617 | Free Cholesterol to Total Lipids in Large LDL percentage | L-LDL-FC/TL (%) |
| 23592 | Free Cholesterol to Total Lipids in Large VLDL percentage | L-VLDL-FC/TL (%) |
| 23642 | Free Cholesterol to Total Lipids in Medium HDL percentage | M-HDL-FC/TL (%) |
| 23622 | Free Cholesterol to Total Lipids in Medium LDL percentage | M-LDL-FC/TL (%) |
| 23597 | Free Cholesterol to Total Lipids in Medium VLDL percentage | M-VLDL-FC/TL (%) |
| 23647 | Free Cholesterol to Total Lipids in Small HDL percentage | S-HDL-FC/TL (%) |
| 23627 | Free Cholesterol to Total Lipids in Small LDL percentage | S-LDL-FC/TL (%) |
| 23602 | Free Cholesterol to Total Lipids in Small VLDL percentage | S-VLDL-FC/TL (%) |
| 23632 | Free Cholesterol to Total Lipids in Very Large HDL percentage | XL-HDL-FC/TL (%) |
| 23587 | Free Cholesterol to Total Lipids in Very Large VLDL percentage | XL-VLDL-FC/TL (%) |
| 23607 | Free Cholesterol to Total Lipids in Very Small VLDL percentage | XS-VLDL-FC/TL (%) |
| 23470 | Glucose | Glucose |
| 23461 | Glutamine | Gln |
| 23462 | Glycine | Gly |
| 23480 | Glycoprotein Acetyls | GlycA |
| 23406 | HDL Cholesterol | HDL-C |
| 23463 | Histidine | His |
| 23465 | Isoleucine | Ile |
| 23471 | Lactate | Lactate |
| 23405 | LDL Cholesterol | LDL-C |
| 23466 | Leucine | Leu |
| 23449 | Linoleic Acid | LA |
| 23456 | Linoleic Acid to Total Fatty Acids percentage | LA/TFA (%) |
| 23447 | Monounsaturated Fatty Acids | MUFA |
| 23454 | Monounsaturated Fatty Acids to Total Fatty Acids percentage | MUFA/TFA (%) |
| 23444 | Omega-3 Fatty Acids | Omega-3 |
| 23451 | Omega-3 Fatty Acids to Total Fatty Acids percentage | Omega-3/TFA (%) |
| 23445 | Omega-6 Fatty Acids | Omega-6 |
| 23459 | Omega-6 Fatty Acids to Omega-3 Fatty Acids ratio | Omega-6/Omega-3 |
| 23452 | Omega-6 Fatty Acids to Total Fatty Acids percentage | Omega-6/TFA (%) |
| 23468 | Phenylalanine | Phe |
| 23437 | Phosphatidylcholines | Phosphatidylc |
| 23434 | Phosphoglycerides | Phosphoglyc |
| 23483 | Phospholipids in Chylomicrons and Extremely Large VLDL | XXL-VLDL-PL |
| 23414 | Phospholipids in HDL | HDL-PL |
| 23525 | Phospholipids in IDL | IDL-PL |
| 23560 | Phospholipids in Large HDL | L-HDL-PL |
| 23532 | Phospholipids in Large LDL | L-LDL-PL |
| 23497 | Phospholipids in Large VLDL | L-VLDL-PL |
| 23413 | Phospholipids in LDL | LDL-PL |
| 23567 | Phospholipids in Medium HDL | M-HDL-PL |
| 23539 | Phospholipids in Medium LDL | M-LDL-PL |
| 23504 | Phospholipids in Medium VLDL | M-VLDL-PL |
| 23574 | Phospholipids in Small HDL | S-HDL-PL |
| 23546 | Phospholipids in Small LDL | S-LDL-PL |
| 23511 | Phospholipids in Small VLDL | S-VLDL-PL |
| 23553 | Phospholipids in Very Large HDL | XL-HDL-PL |
| 23490 | Phospholipids in Very Large VLDL | XL-VLDL-PL |
| 23518 | Phospholipids in Very Small VLDL | XS-VLDL-PL |
| 23412 | Phospholipids in VLDL | VLDL-PL |
| 23579 | Phospholipids to Total Lipids in Chylomicrons and Extremely Large VLDL percentage | XXL-VLDL-PL/TL (%) |
| 23609 | Phospholipids to Total Lipids in IDL percentage | IDL-PL/TL (%) |
| 23634 | Phospholipids to Total Lipids in Large HDL percentage | L-HDL-PL/TL (%) |
| 23614 | Phospholipids to Total Lipids in Large LDL percentage | L-LDL-PL/TL (%) |
| 23589 | Phospholipids to Total Lipids in Large VLDL percentage | L-VLDL-PL/TL (%) |
| 23639 | Phospholipids to Total Lipids in Medium HDL percentage | M-HDL-PL/TL (%) |
| 23619 | Phospholipids to Total Lipids in Medium LDL percentage | M-LDL-PL/TL (%) |
| 23594 | Phospholipids to Total Lipids in Medium VLDL percentage | M-VLDL-PL/TL (%) |
| 23644 | Phospholipids to Total Lipids in Small HDL percentage | S-HDL-PL/TL (%) |
| 23624 | Phospholipids to Total Lipids in Small LDL percentage | S-LDL-PL/TL (%) |
| 23599 | Phospholipids to Total Lipids in Small VLDL percentage | S-VLDL-PL/TL (%) |
| 23629 | Phospholipids to Total Lipids in Very Large HDL percentage | XL-HDL-PL/TL (%) |
| 23584 | Phospholipids to Total Lipids in Very Large VLDL percentage | XL-VLDL-PL/TL (%) |
| 23604 | Phospholipids to Total Lipids in Very Small VLDL percentage | XS-VLDL-PL/TL (%) |
| 23446 | Polyunsaturated Fatty Acids | PUFA |
| 23458 | Polyunsaturated Fatty Acids to Monounsaturated Fatty Acids ratio | PUFA/MUFA |
| 23453 | Polyunsaturated Fatty Acids to Total Fatty Acids percentage | PUFA/TFA (%) |
| 23472 | Pyruvate | Pyruvate |
| 23402 | Remnant Cholesterol (Non-HDL, Non-LDL -Cholesterol) | Remnant-C |
| 23448 | Saturated Fatty Acids | SFA |
| 23455 | Saturated Fatty Acids to Total Fatty Acids percentage | SFA/TFA (%) |
| 23438 | Sphingomyelins | Sphingomyelins |
| 23400 | Total Cholesterol | Total-C |
| 23401 | Total Cholesterol Minus HDL-C | non-HDL-C |
| 23436 | Total Cholines | Cholines |
| 23464 | Total Concentration of Branched-Chain Amino Acids (Leucine + Isoleucine + Valine) | Total BCAA |
| 23427 | Total Concentration of Lipoprotein Particles | Total-P |
| 23415 | Total Esterified Cholesterol | Total-CE |
| 23442 | Total Fatty Acids | Total-FA |
| 23419 | Total Free Cholesterol | Total-FC |
| 23482 | Total Lipids in Chylomicrons and Extremely Large VLDL | XXL-VLDL-L |
| 23426 | Total Lipids in HDL | HDL-L |
| 23524 | Total Lipids in IDL | IDL-L |
| 23559 | Total Lipids in Large HDL | L-HDL-L |
| 23531 | Total Lipids in Large LDL | L-LDL-L |
| 23496 | Total Lipids in Large VLDL | L-VLDL-L |
| 23425 | Total Lipids in LDL | LDL-L |
| 23423 | Total Lipids in Lipoprotein Particles | Total-L |
| 23566 | Total Lipids in Medium HDL | M-HDL-L |
| 23538 | Total Lipids in Medium LDL | M-LDL-L |
| 23503 | Total Lipids in Medium VLDL | M-VLDL-L |
| 23573 | Total Lipids in Small HDL | S-HDL-L |
| 23545 | Total Lipids in Small LDL | S-LDL-L |
| 23510 | Total Lipids in Small VLDL | S-VLDL-L |
| 23552 | Total Lipids in Very Large HDL | XL-HDL-L |
| 23489 | Total Lipids in Very Large VLDL | XL-VLDL-L |
| 23517 | Total Lipids in Very Small VLDL | XS-VLDL-L |
| 23424 | Total Lipids in VLDL | VLDL-L |
| 23411 | Total Phospholipids in Lipoprotein Particles | Total-PL |
| 23407 | Total Triglycerides | Total-TG |
| 23487 | Triglycerides in Chylomicrons and Extremely Large VLDL | XXL-VLDL-TG |
| 23410 | Triglycerides in HDL | HDL-TG |
| 23529 | Triglycerides in IDL | IDL-TG |
| 23564 | Triglycerides in Large HDL | L-HDL-TG |
| 23536 | Triglycerides in Large LDL | L-LDL-TG |
| 23501 | Triglycerides in Large VLDL | L-VLDL-TG |
| 23409 | Triglycerides in LDL | LDL-TG |
| 23571 | Triglycerides in Medium HDL | M-HDL-TG |
| 23543 | Triglycerides in Medium LDL | M-LDL-TG |
| 23508 | Triglycerides in Medium VLDL | M-VLDL-TG |
| 23578 | Triglycerides in Small HDL | S-HDL-TG |
| 23550 | Triglycerides in Small LDL | S-LDL-TG |
| 23515 | Triglycerides in Small VLDL | S-VLDL-TG |
| 23557 | Triglycerides in Very Large HDL | XL-HDL-TG |
| 23494 | Triglycerides in Very Large VLDL | XL-VLDL-TG |
| 23522 | Triglycerides in Very Small VLDL | XS-VLDL-TG |
| 23408 | Triglycerides in VLDL | VLDL-TG |
| 23435 | Triglycerides to Phosphoglycerides ratio | TG/Phosphoglycerides |
| 23583 | Triglycerides to Total Lipids in Chylomicrons and Extremely Large VLDL percentage | XXL-VLDL-TG/TL (%) |
| 23613 | Triglycerides to Total Lipids in IDL percentage | IDL-TG/TL (%) |
| 23638 | Triglycerides to Total Lipids in Large HDL percentage | L-HDL-TG/TL (%) |
| 23618 | Triglycerides to Total Lipids in Large LDL percentage | L-LDL-TG/TL (%) |
| 23593 | Triglycerides to Total Lipids in Large VLDL percentage | L-VLDL-TG/TL (%) |
| 23643 | Triglycerides to Total Lipids in Medium HDL percentage | M-HDL-TG/TL (%) |
| 23623 | Triglycerides to Total Lipids in Medium LDL percentage | M-LDL-TG/TL (%) |
| 23598 | Triglycerides to Total Lipids in Medium VLDL percentage | M-VLDL-TG/TL (%) |
| 23648 | Triglycerides to Total Lipids in Small HDL percentage | S-HDL-TG/TL (%) |
| 23628 | Triglycerides to Total Lipids in Small LDL percentage | S-LDL-TG/TL (%) |
| 23603 | Triglycerides to Total Lipids in Small VLDL percentage | S-VLDL-TG/TL (%) |
| 23633 | Triglycerides to Total Lipids in Very Large HDL percentage | XL-HDL-TG/TL (%) |
| 23588 | Triglycerides to Total Lipids in Very Large VLDL percentage | XL-VLDL-TG/TL (%) |
| 23608 | Triglycerides to Total Lipids in Very Small VLDL percentage | XS-VLDL-TG/TL (%) |
| 23469 | Tyrosine | Tyr |
| 23467 | Valine | Val |
| 23403 | VLDL Cholesterol | VLDL-C |

**Supplementary Figure 2.** Directed acyclic graph for variable selection.

**Supplementary Table 2.** Characteristics of the study populations by missing covariate status.

| Characteristics | Missing any covariates (n = 41,942) | Complete  (n = 409,980) |
| --- | --- | --- |
| Age (year) | 58.5 ± 7.8 | 56.4 ± 8.1 |
| Female | 25253 (60.2) | 218874 (53.4) |
| Education (college) | 6296 (16.8) | 143873 (35.1) |
| PRS_PD_ | -0.2 (-0.8, 0.5) | -0.2 (-0.8, 0.5) |
| Depressive symptoms | 0 (0, 1) | 0 (0, 1) |
| Incident of Parkinson’s disease | 413 (0.9) | 2695 (0.7) |

Data were presented as mean ± standard deviation for continuous variables and number (percentage) for categorical variables.

Abbreviations: PRS_PD_ = polygenic risk score for Parkinson’s disease.

**Supplementary Table 3.** Hazard ratios (HRs) and 95% confidence intervals (CIs) for the joint effect of depressive symptoms and genetic risk factors on the risk of incident Parkinson’s disease.

| **Joint effect** | | ***No. of participants*** | **Parkinson’s disease** | | |
| --- | --- | --- | --- | --- | --- |
|  |  |  | No. of cases (%) | HR (95% CI) ^a^ | HR (95% CI) ^b^ |
| **Depressive disorder** | **PRS_PD_** |  |  |  |  |
| *No* | *Low* | 141,805 | 663 (0.47) | Reference | Reference |
| *Yes* | *Low* | 8,836 | 57 (0.65) | 2.03 (1.55, 2.67) | 1.86 (1.37, 2.53) |
| *No* | *High* | 141,667 | 1,356 (0.96) | 1.73 (1.58, 1.88) | 1.74 (1.59, 1.91) |
| *Yes* | *High* | 8,962 | 82 (0.91) | 2.40 (1.99, 2.89) | 2.15 (1.74, 2.65) |
| *P for interaction* | |  |  | 0.021 | 0.028 |
| **Stratification analysis** | |  |  |  |  |
| PRS_PD_ | |  |  |  |  |
| Low | | 150,641 | 720 (0.48) | 2.07 (1.57, 2.72) | 1.72 (1.25, 2.38) |
| High | | 150,639 | 1,438 (0.95) | 1.37 (1.09, 1.72) | 1.31 (1.02, 1.69) |

^a^ Models were adjusted for age, sex, and education.

^b^ Models were adjusted for age, sex, education, race, socioeconomic status, body mass index, smoking status, alcohol drinking, physical activity, social activity, sleep duration, hypertension, diabetes, and cardiovascular disease.

Abbreviations: PRS_PD_ = polygenic risk score for Parkinson’s disease.

**Supplementary Table 4.** Mediation effect of plasma metabolomic signature on the associations between pre-clinical depressive symptoms and incident Parkinson’s disease.

| Effect | Items |  | Coefficients |  |
| --- | --- | --- | --- | --- |
|  |  |  | β (95% CI) | *P*-value |
| Direct effect | Depressive symptoms → Parkinson’s disease |  | 0.28 (0.19, 0.43) | 0.003 |
| Indirect effect | Depressive symptoms → Metabolomic signature |  | 0.10 (0.07, 0.15) | 0.001 |
|  | Metabolomic signature → Parkinson’s disease |  | 0.40 (0.24, 0.53) | <0.001 |
| Total effect | Depressive symptoms → Parkinson’s disease |  | 0.24 (0.12, 0.40) | 0.021 |
| ***Mediation proportion (%)*** | | | 14.95 (5.53, 30.24) | 0.008 |

Models adjusted for age, sex, education, race, socioeconomic status, body mass index, smoking status, alcohol drinking, physical activity, social activity, sleep duration, hypertension, diabetes, and cardiovascular disease.

Bootstrap was used to estimate the confidence intervals, with 500 iterations.

**Supplementary Table 5.** Mediation effect of plasma metabolomic signature (PCA) on the associations between pre-clinical depressive symptoms and incident Parkinson’s disease.

| Effect | Items |  | Coefficients |  |
| --- | --- | --- | --- | --- |
|  |  |  | β (95% CI) | *P*-value |
| Direct effect | Depressive symptoms → Parkinson’s disease |  | 0.25 (0.08, 0.45) | 0.013 |
| Indirect effect | Depressive symptoms → Metabolomic signature |  | 0.17 (0.14, 0.19) | <0.001 |
|  | Metabolomic signature → Parkinson’s disease |  | 0.27 (0.10, 0.33) | <0.001 |
| Total effect | Depressive symptoms → Parkinson’s disease |  | 0.30 (0.15, 0.51) | 0.045 |
| ***Mediation proportion (%)*** | | | 22.11 (13.78, 29.42) | 0.011 |

Models adjusted for age, sex, education, race, socioeconomic status, body mass index, smoking status, alcohol drinking, physical activity, social activity, sleep duration, hypertension, diabetes, and cardiovascular disease.

Bootstrap was used to estimate the confidence intervals, with 500 iterations.

**Supplementary Table 6.** Hazard ratios (HRs) and 95% confidence intervals (CIs) for incident Parkinson’s disease in relation to depressive symptoms: stratified analysis.

| **Depressive symptoms** | **No. of participants** | **Parkinson’s disease** | | |
| --- | --- | --- | --- | --- |
|  |  | **No. of cases** | **HR (95% CI) ^a^** | **HR (95% CI) ^b^** |
| Sex |  |  |  |  |
| Female | 244,127 | 1,150 | 1.12 (1.06, 1.17) | 1.10 (1.04, 1.16) |
| Male | 207,795 | 1,958 | 1.15 (1.11, 1.20) | 1.10 (1.06, 1.16) |
| Interaction | - | - | 1.03 (0.96, 1.09)  *P*=0.414 | 0.99 (0.93, 1.07)  *P*=0.868 |
| Age |  |  |  |  |
| <65 years | 365,614 | 1,629 | 1.16 (1.12, 1.21) | 1.12 (1.07, 1.17) |
| ≥65 years | 86,308 | 1,479 | 1.10 (1.04, 1.16) | 1.07 (1.01, 1.14) |
| Interaction | - | - | 0.95 (0.89, 1.01)  *P*=0.119 | 0.95 (0.88, 1.02)  *P*=0.150 |
| Physical activity |  |  |  |  |
| Regular | 302,372 | 1,961 | 1.54 (1.27, 1.89) | 1.45 (1.18, 1.78) |
| Irregular | 117,156 | 827 | 1.27 (0.96, 1.69) | 1.14 (0.85, 1.53) |
| Interaction | - | - | 1.18 (0.84, 1.67)  *P*=0.341 | 0.84 (0.60, 1.19)  *P*=0.325 |
| Sleep duration |  |  |  |  |
| 6-8 hours | 340,744 | 2,350 | 1.52 (1.18, 1.95) | 1.40 (1.06, 1.86) |
| <6 hours or >8 hours | 109,139 | 736 | 1.55 (1.29, 1.87) | 1.33 (1.08, 1.64) |
| Interaction | - | - | 1.04 (0.75, 1.41)  *P*=0.827 | 0.96 (0.68, 1.36)  *P*=0.817 |
| Cardiovascular disease |  |  |  |  |
| Yes | 30,384 | 484 | 1.42 (1.03, 1.97) | 1.32 (0.90, 1.92) |
| No | 421,838 | 2,624 | 1.50 (1.27, 1.77) | 1.34 (1.11, 1.63) |
| Interaction | - | - | 1.01 (0.70, 1.45)  *P*=0.968 | 1.03 (0.68, 1.55)  *P*=0.899 |
| Diabetes |  |  |  |  |
| Yes | 23,420 | 336 | 1.49 (1.04, 2.13) | 1.25 (0.81, 1.91) |
| No | 428,502 | 2,772 | 1.48 (1.26, 1.74) | 1.36 (1.13, 1.63) |
| Interaction | - | - | 0.95 (0.64, 1.40)  *P*=0.782 | 0.96 (0.61, 1.52)  *P*=0.860 |

^a^ Models were adjusted for age, sex, and education.

^b^ Models were adjusted for age, sex, education, race, socioeconomic status, body mass index, smoking status, alcohol drinking, physical activity, social activity, sleep duration, hypertension, diabetes, cardiovascular disease, and PRS_PD_.

**Supplementary Table 7.** Hazard ratios (HRs) and 95% confidence intervals (CIs) for incident Parkinson’s disease in relation to depressive symptoms: after excluding participants younger than 50 years at baseline.

| **Depressive symptoms** | **No. of participants** | **Parkinson’s disease** | | |
| --- | --- | --- | --- | --- |
|  |  | **No. of cases** | **HR (95% CI) ^a^** | **HR (95% CI) ^b^** |
| Continuous | 346,762 | 3,028 | 1.13 (1.10, 1.17) | 1.10 (1.06, 1.14) |
| Categorical |  |  |  |  |
| No depression | 328,446 | 2,847 | Reference | Reference |
| Possible depressive disorder | 18,316 | 181 | 1.50 (1.228, 1.74) | 1.32 (1.12, 1.58) |

^a^ Models were adjusted for age, sex, and education.

^b^ Models were adjusted for age, sex, education, race, socioeconomic status, body mass index, smoking status, alcohol drinking, physical activity, social activity, sleep duration, hypertension, diabetes, cardiovascular disease, and PRS_PD_.

**Supplementary Table 8.** Hazard ratios (HRs) and 95% confidence intervals (CIs) for incident Parkinson’s disease in relation to depressive symptoms: after excluding participants with a history of clinically diagnosed depression.

| **Depressive symptoms** | **No. of participants** | **Parkinson’s disease** | | |
| --- | --- | --- | --- | --- |
|  |  | **No. of cases** | **HR (95% CI) ^a^** | **HR (95% CI) ^b^** |
| Continuous | 415,929 | 2,818 | 1.11 (1.07, 1.15) | 1.08 (1.03, 1.13) |
| Categorical |  |  |  |  |
| No depression | 396,834 | 2,695 | Reference | Reference |
| Possible depressive disorder | 19,095 | 123 | 1.31 (1.09, 1.58) | 1.23 (1.02, 1.49) |

^a^ Models were adjusted for age, sex, and education.

^b^ Models were adjusted for age, sex, education, race, socioeconomic status, body mass index, smoking status, alcohol drinking, physical activity, social activity, sleep duration, hypertension, diabetes, cardiovascular disease, and PRS_PD_.

**Supplementary Table 9.** Incidence rate ratio (IRRs) and 95% confidence intervals (CIs) for incident Parkinson’s disease in relation to depressive symptoms: results from Poisson regression.

| **Depressive symptoms** | **No. of participants** | **Parkinson’s disease** | | |
| --- | --- | --- | --- | --- |
|  |  | **No. of cases** | **IRR (95% CI) ^a^** | **IRR (95% CI) ^b^** |
| Continuous | 451,922 | 3,108 | 1.15 (1.12, 1.19) | 1.11 (1.07, 1.15) |
| Categorical |  |  |  |  |
| No depression | 425,179 | 2,914 | Reference | Reference |
| Possible depressive disorder | 26,743 | 194 | 1.61 (1.39, 1.86) | 1.35 (1.14, 1.60) |

^a^ Models were adjusted for age, sex, and education.

^b^ Models were adjusted for age, sex, education, race, socioeconomic status, body mass index, smoking status, alcohol drinking, physical activity, social activity, sleep duration, hypertension, diabetes, cardiovascular disease, and PRS_PD_.

**Supplementary Table 10.** Hazard ratios (HRs) and 95% confidence intervals (CIs) for incident Parkinson’s disease in relation to depressive symptoms: after excluding incident Parkinson’s disease cases during the first 3 years of follow-up.

| **Depressive symptoms** | **No. of participants** | **Parkinson’s disease** | | |
| --- | --- | --- | --- | --- |
|  |  | **No. of cases** | **HR (95% CI) ^a^** | **HR (95% CI) ^b^** |
| Continuous | 451,795 | 2,981 | 1.12 (1.09, 1.16) | 1.09 (1.05, 1.13) |
| Categorical |  |  |  |  |
| No depression | 425,066 | 2,801 | Reference | Reference |
| Possible depressive disorder | 26,729 | 180 | 1.19 (1.02, 1.39) | 1.30 (1.09, 1.54) |

^a^ Models were adjusted for age, sex, and education.

^b^ Models were adjusted for age, sex, education, race, socioeconomic status, body mass index, smoking status, alcohol drinking, physical activity, social activity, sleep duration, hypertension, diabetes, cardiovascular disease, and PRS_PD_.

**Supplementary Table 11.** Hazard ratios (HRs) and 95% confidence intervals (CIs) for incident Parkinson’s disease in relation to depressive symptoms: after excluding incident Parkinson’s disease cases during the first 5 years of follow-up.

| **Depressive symptoms** | **No. of participants** | **Parkinson’s disease** | | |
| --- | --- | --- | --- | --- |
|  |  | **No. of cases** | **HR (95% CI) ^a^** | **HR (95% CI) ^b^** |
| Continuous | 451,565 | 3,108 | 1.12 (1.08, 1.16) | 1.09 (1.05, 1.13) |
| Categorical |  |  |  |  |
| No depression | 424,842 | 2,914 | Reference | Reference |
| Possible depressive disorder | 26,684 | 194 | 1.18 (1.01, 1.39) | 1.28 (1.06, 1.53) |

^a^ Models were adjusted for age, sex, and education.

^b^ Models were adjusted for age, sex, education, race, socioeconomic status, body mass index, smoking status, alcohol drinking, physical activity, social activity, sleep duration, hypertension, diabetes, cardiovascular disease, and PRS_PD_.

**Supplementary Table 12.** Hazard ratios (HRs) and 95% confidence intervals (CIs) for incident Parkinson’s disease in relation to depressive symptoms: after multiple imputation of missing values of covariates.

| **Depressive symptoms** | **No. of participants** | **Parkinson’s disease** | | |
| --- | --- | --- | --- | --- |
|  |  | **No. of cases** | **HR (95% CI) ^a^** | **HR (95% CI) ^b^** |
| Continuous | 451,922 | 3,108 | 1.14 (1.10, 1.17) | 1.12 (1.08, 1.15) |
| Categorical |  |  |  |  |
| No depression | 425,179 | 2,914 | Reference | Reference |
| Possible depressive disorder | 26,743 | 194 | 1.53 (1.32, 1.78) | 1.42 (1.22, 1.65) |

^a^ Models were adjusted for age, sex, and education.

^b^ Models were adjusted for age, sex, education, race, socioeconomic status, body mass index, smoking status, alcohol drinking, physical activity, social activity, sleep duration, hypertension, diabetes, cardiovascular disease, and PRS_PD_.

**Supplementary Table 13.** Hazard ratios (HRs) and 95% confidence intervals (CIs) for incident Parkinson’s disease in relation to depressive symptoms: after excluding 396 participants with other Parkinsonian syndromes at baseline or follow-up.

| **Depressive symptoms** | **No. of participants** | **Parkinson’s disease** | | |
| --- | --- | --- | --- | --- |
|  |  | **No. of cases** | **HR (95% CI) ^a^** | **HR (95% CI) ^b^** |
| Continuous | 451,526 | 2,751 | 1.14 (1.10, 1.18) | 1.11 (1.07, 1.15) |
| Categorical |  |  |  |  |
| No depression | 424,851 | 2,586 | Reference | Reference |
| Possible depressive disorder | 26,714 | 165 | 1.54 (1.32, 1.78) | 1.37 (1.15, 1.62) |

^a^ Models were adjusted for age, sex, and education.

^b^ Models were adjusted for age, sex, education, race, socioeconomic status, body mass index, smoking status, alcohol drinking, physical activity, social activity, sleep duration, hypertension, diabetes, cardiovascular disease, and PRS_PD_.

**Supplementary Table 14.** Hazard ratios (HRs) and 95% confidence intervals (CIs) for incident Parkinson’s disease in relation to depressive symptoms: with age time time-scale in the Cox regressions.

| **Depressive symptoms** | **No. of participants** | **Parkinson’s disease** | | |
| --- | --- | --- | --- | --- |
|  |  | **No. of cases** | **HR (95% CI) ^a^** | **HR (95% CI) ^b^** |
| Continuous | 451,922 | 3,108 | 1.14 (1.10, 1.18) | 1.11 (1.08, 1.15) |
| Categorical |  |  |  |  |
| No depression | 425,179 | 2,914 | Reference | Reference |
| Possible depressive disorder | 26,743 | 194 | 1.54 (1.33, 1.78) | 1.34 (1.13, 1.59) |

^a^ Models were adjusted for age, sex, and education.

^b^ Models were adjusted for age, sex, education, race, socioeconomic status, body mass index, smoking status, alcohol drinking, physical activity, social activity, sleep duration, hypertension, diabetes, cardiovascular disease, and PRS_PD_.
